# The Senescence of Cut Daffodil Flowers Correlates with Programmed Cell Death Symptoms

**DOI:** 10.3390/ijms26157657

**Published:** 2025-08-07

**Authors:** Julita Rabiza-Świder, Sutrisno, Piotr Salachna, Agnieszka Zawadzińska, Ewa Skutnik

**Affiliations:** 1 Section of Ornamental Plants, Institute of Horticultural Sciences, Warsaw University of Life Sciences, Nowoursynowska 166, 02-787 Warsaw, Poland; sutrisno_sutrisno@sggw.edu.pl (S.); ewa_skutnik@sggw.edu.pl (E.S.); 2 Department of Horticulture, West Pomeranian University of Technology in Szczecin, Słowackiego 17, 71-434 Szczecin, Poland; piotr.salachna@zut.edu.pl (P.S.); agnieszka.zawadzinska@zut.edu.pl (A.Z.)

**Keywords:** catalase, corona, oxidative stress, perianth, proline, sugars

## Abstract

Daffodils are among the most popular bulbous plants for cut flowers, especially Trumpet cultivars. The aim of this study was to evaluate changes in cut daffodil flowers and to determine the response of perianth senescence in cut daffodil flowers in a different way than the corona does and to determine whether the senescence of cut daffodil flowers is correlated with PCD symptoms. During the senescence of cut daffodil flowers, there was an increase in free proline, malondialdehyde and hydrogen peroxide contents and increased catalase activity. Typically, senescence processes occurred faster in the perianth than in the corona, excluding carbohydrates, which had a higher content in the perianth than in the corona. One of the symptoms of daffodil flower senescence was the degradation of cell nuclei. In addition, chromatin fragmentation could also be observed in the corona. The nuclei in the perianth began to change their spherical shape and decay. In the corona, the nuclear envelope retained its continuity much longer and started to disintegrate later than in the perianth. This is possibly because the corona has a longer vase life than the perianth.

## 1. Introduction

Daffodils are among the most popular bulbous plants and are most popularly chosen just after tulips and lilies. They are particularly popular in spring, which is when they flower in the wild. However, individuals can also obtain cut daffodils grown under a cover from as early as December. The most common and the most popular cut flowers are the Trumpet daffodil cultivars.

Several factors affect the longevity of daffodil flowers, such as genetic factors, growing conditions, harvest stage and postharvest treatment [1].

Flower senescence is a highly organized process leading to its death, involving biochemical, molecular and structural changes [2,3]. It is regulated by a variety of factors, but the death of individual tissues and cells within the flower is coordinated at multiple levels [4]. During senescence, processes typical of apoptotic cells, such as chromatin condensation, DNA fragmentation and cell nucleus breakdown, are observed. It consists of a series of programmed phenomena leading to the degradation and remobilization of proteins, lipids and nucleic acids. This process complies with the general definition of programmed cell death (PCD) [5]. According to Shibuya et al. [6], PCD is connected with the death of individual cells and senescence in the context of the development of whole organs, including the nutrient remobilization process. The term PCD describes cell death, which is a part of the normal life cycle of organisms and refers to the process in which cells are eliminated within a developmental or adaptive event in the life cycle of an organism [7]. The beginning and course of PCD in plants can be observed under a microscope, as has been performed in flowers of the iris (*Iris*) [8], Peruvian lily (*Alstroemeria hybrida*) [9], *Gypsophila paniculata* [7], lilac (*Syringa vulgaris*) [10], *Clematis* [11] and snapdragon (*Antirrhinum majus*) [12]. Shrinking of the nucleus in Peruvian lily petals was visible at the same time as the symptoms of senescence appeared [9], whereas in *Gypsophila*, mesophyll cells collapsed even before the signs of senescence became visible [7]. According to van Doorn et al. [8], changes in the ultrastructures of aging petals occur before symptoms are visible. In both plants (*Alstroemeria* and *Gypsophila*), signs of senescence were visibly correlated with the degeneration of epidermis cells. PCD in petals causes various visible senescence symptoms, such as wilting or color changes [6].

The model ornamental plant on which PCD has been studied is the daylily (*Hemerocallis hybrida*), whose flowers persist for only 24 h [13]. The appearance of symptoms of petal cell death is preceded by massive ion leakage due to the disorganization of cytoplasmic membranes and the loss of semipermeable properties. Van Doorn et al. [8] confirmed that most of the biochemical and ultrastructural changes in iris petals occur at a very early stage of flower development, shortly after bud opening. The individual vacuoles are then merged into one large vacuole, which fills the whole cell. The activity of phospholipase D also increases, and on successive days of flower development, the activity of proteolytic enzymes increases, and cell swelling and gradual degradation occur, although the symptoms of petal senescence are yet invisible. Petal PCD also involves several other morphological features associated with autolytic PCD, including chromatin condensation in snapdragon, *Argyranthemum* and *Petunia* [14] and an increase in vacuolar volume in *Iris* [8]. In the *Iris* flag tepals, condensation of chromatin takes place at a rather late stage of senescence, when the flag tepals roll inside, which is also accompanied by total cell degradation and death.

During the senescence process, macromolecular compounds such as proteins, nucleic acids, pigments, lipids and polysaccharides of cell walls break down, and this degradation is caused by the synthesis or increase in activity of hydrolytic enzymes such as proteases, nucleases, amylases, pectinases and cellulases, whose production frequently depends on the activation of certain genes [15,16]. Degradation of cell walls is believed to contribute to cell death and is accompanied by turgor loss and cell collapse together with tonoplast rupture. Shibyua et al. [6] reported cell wall degradation during petal PCD. In cut flowers, water stress enhances this senescence process, causing several changes to the nitrogen metabolism, among other processes, leading to ammonium and free proline accumulation; depletion of the respiratory substrate, mainly carbohydrates, which limits the energy available to sustain life processes; lipid peroxidation, connected with malondialdehyde (MDA) accumulation; and harmful effects of reactive oxygen species (ROS) emerging during oxidative stress. Reactive oxygen species such as hydrogen peroxide are generated, damaging cells and hastening the death of cells and, consequently, the entire organism. All these biochemical factors can lead to cell death and also play a role in senescing cut daffodil flowers.

In the daffodil senescence process, first, the flowers lose their turgor, then the petal tips dry up, and only then does the corolla, which also loses vigor and falls off in the final stage of senescence. The longevity of the daffodil perianth varies from 4 to 5 days, whereas that of the corona varies from 6 to 7 days [1,17], depending on the cultivar. After 32 daffodil cultivars were tested, Hunter and Reid [18] reported that flower life varies from approximately 8 days in the cultivar ‘Investment’ to 13 days in the cultivar ‘Tibet’. The difference in the longevity of the perianth and corona may be due to different rates and differences in the senescence process of these structures, so the purpose of this study is to compare the senescence process of the perianth and corona of cut daffodil ‘Dutch Master’ flowers.

Studies on the PCD of flowers indicate that early changes in the structure of petals and the ultrastructures of their cells often occur before visible symptoms of senescence appear. Shibuya and co-workers [6] claim that the elucidation of the underlying biochemical mechanisms behind the morphological changes in petal PCD is one of the major challenges in the studies of petal senescence. The aim of this study was to evaluate these changes in cut daffodil flowers and determine whether the response to perianth senescence differs from that of the corona in cut daffodil flowers and whether the senescence of cut daffodil flowers is correlated with biochemical changes (sugars, free proline, malondialdehyde, hydrogen peroxide contents, and catalase activity) and nuclear degradation as PCD symptoms.

## 2. Results

In the ‘Dutch Master’ flowers, the content of total sugars in the perianth decreased from the harvest day until the fourth day (Table 1). In the last two days (days 5 and 6), it increased significantly, reaching its highest value on day 6. On these two dates (days 5 and 6), it was also significantly higher than the level in the corona. The content of total sugars in the corona was quite different from that in the perianth. Until the third day, when the highest value was recorded (higher than in the perianth too), the level of total sugars increased significantly. Then, it started to decrease, and on the last two days, it remained constant. The content of total sugars in the perianth on the last day was 17% greater than that on the day of harvest, whereas in the coronas, it was 13% lower.

The highest content of reducing sugars in the perianth was recorded on the second day after harvest (Table 1). This content was twice as high as that on the harvest day; however, it did not differ significantly from the content on the third and fifth days. The lowest content of reducing sugars in both the perianth and corona was recorded on the harvest day and was almost twice as low as that on the last day of the experiment.

The content of free proline both in the perianth and in the corolla increased significantly every day, reaching up to 6 times higher on the last day than on the day of harvest (Table 1).

The MDA content increased significantly in both the perianth and corona of the cut daffodils ‘Dutch Master’ (Table 1). In the perianth, the highest value was recorded on the second and fifth days after harvest. In the corona, the highest MDA content was detected on the third and fifth days. In both cases, this level reached approximately 140–150 nmol·g^−1^DW.

In the perianth, the hydrogen peroxide content remained at a similar level until the fourth day (Table 1). It increased significantly over the last two days, reaching its highest value on day six. In the corona, the H_2_O_2_ content gradually increased and then decreased. On the last day of the experiment, it was as much as 44% lower than that on the harvest day. From the first day after harvesting, the hydrogen peroxide content was significantly higher in the perianth than in the corona.

The highest catalase activity in the perianth was recorded on the sixth day after harvest (Table 1). This value was as much as 5 times greater than that on the harvest day. In the corona, on the other hand, the catalase activity on the last day was as much as 8 times greater than that on the harvest day and differed considerably from the activity on the other days.

In the cut ‘Mando’ flowers, the content of total sugars in both the perianth and the corona increased on the first day after harvest, thus reaching the highest value (Table 2). On the following days, it alternately fluctuated, increasing and then decreasing. On the last day, the content of soluble sugars in the perianth was 21% lower than that on the day of flower harvest. A similar situation occurred in the corona, but on the last (seventh) day, it was almost half (47%) of that on the harvest day. On most measurement days (6 out of 8), the content of total sugars was higher in the perianth leaves than in the corona.

The analysis of variance revealed a significant relationship between the reducing sugar content on individual days (Table 2). In the perianth, the level of reducing sugars reached its lowest value on the first day after harvest. The third day presented the highest value, which was twice as high as that on the harvest day. In the following days, the content of reducing sugars in the perianth gradually decreased. A similar situation occurred in the corona, but in this case, the highest content of reducing sugars was reached on the second day of the experiment. In this case, from day 3, the reducing sugar content was also lower than that in the perianth.

The content of free proline in the perianth and corona was lower on the first day after harvest than on the harvest day, but it increased significantly each following day (Table 2). On the last day of the measurement in the perianth, it was 3 times greater than that at the beginning, whereas in the corona, it was as much as 4 times greater. On five of the eight measurement dates (days 0, 3, 4, 5, 6), the free proline content was higher in the perianth than in the corona.

The MDA content increased significantly, both in the perianth and corona of cut daffodils ‘Mando’ (Table 2). In the perianth and corona at the end of the experiment (day 7), the MDA content was approximately 23% higher than that after harvest. In the perianth, the highest value was recorded on the second, fifth, sixth and seventh days after harvest. In the corona, the highest MDA content was detected on the third and fifth days. In both cases, this level reached approximately 150–170 nmol·g^−1^DW. On most measurement dates (5 out of 8), MDA levels were higher in the perianth than in the corona.

The hydrogen peroxide content in the perianth was the highest on the harvest day and on the last day of the experiment and differed significantly from the amount on the other days, where it remained constant at a lower level (Table 2). The corona H_2_O_2_ content was the highest on the harvest day. On the following days, it fluctuated, reaching the lowest value on day six, which was 1.5 times lower than that on the harvest days. Comparing hydrogen peroxide levels in the perianth and corona, on six of the eight measurement dates (days 0, 1, 3, 5, 6, 7), the content was higher in perianth leaves than in the corona.

The highest catalase activity in the perianth was recorded on the last day, which was almost three times greater than that on the harvest day (Table 2). In the corona, on the other hand, the highest catalase activity was observed on the second day after harvest, and it was as much as four times greater than that on the first day. From the third to the seventh days, it remained at a constant high level. Generally, the catalase activity was higher in the corona than in the perianth on most of the measurement dates.

**Figure 1:**
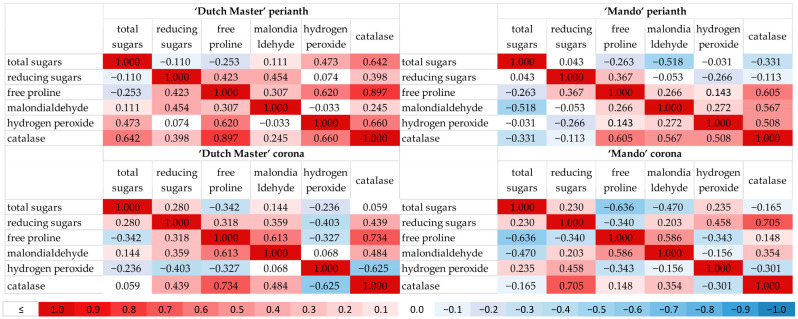
Pearson correlation matrix describing correlations among biochemical parameters in cut daffodil flowers. Marked correlations are significant at *p* < 0.05, presented on a color scale: 0.1–0.29—very low; 0.30–0.49—low; 0.50–0.69—restrained; 0.70–0.89—high; >0.90—very high.

The Pearson correlation coefficients between the tested parameters in the perianth and corona of ‘Dutch Master’ and ‘Mando’ daffodils were also examined (Figure 1). The analysis showed multiple relationships with different strengths between the contents of total sugars, reducing sugars, free proline, malondialdehyde, hydrogen peroxide, and catalase activity.

A positive restrained correlation was observed between the total sugar content and catalase activity, but only in the perianth of ‘Dutch Master’ flowers (Figure 1). Total sugar correlates negatively with free proline content: a higher sugar content reduced the growth of proline, with a stronger power in coronas than in perianths. In contrast, in coronas, catalase activity exhibited negative correlations with hydrogen peroxide contents. This relationship shows that lower oxidative stress markers (hydrogen peroxide) correspond with higher activity of antioxidant enzymes. The inverse situation was observed in perianths of both cultivars tested, where a high level of hydrogen peroxide corresponded with high catalase activity. In perianths, the high levels of hydrogen peroxide also corresponded with high levels of proline, considered a primary indicator of stress. In coronas, this correlation was negative but at a small or very small level.

A high positive correlation was observed between free proline content and catalase activity (Figure 1). Additionally, free proline exhibited a correlation with malondialdehyde content, which was stronger in coronas than in perianths of both cultivars tested. In addition, catalase activity correlated with MDA levels, but at rather low levels.

Sometimes the response of the tested cultivars was stronger. In ‘Mando’, the negative restrained correlation between MDA and total sugars content was stronger than in ‘Dutch Master’ flowers. In addition to the above-mentioned correlations, many others of weaker strength were found in cut ‘Dutch Master’ and ‘Mando’ daffodil flowers.

**Figure 2:**
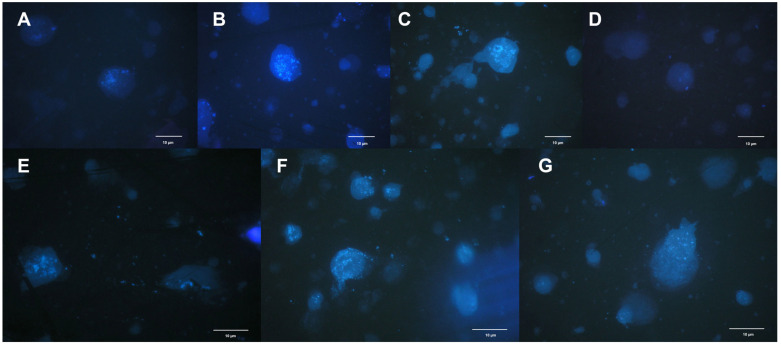
Nuclei degradation in the perianth of cut ‘Dutch Master’ daffodils, collected from flowers (**A**) immediately after harvest, (**B**) 1 day after harvest, (**C**) 2 days after harvest, (**D**) 3 days after harvest, (**E**) 4 days after harvest, (**F**) 5 days after harvest, (**G**) 6 days after harvest.

**Figure 3:**
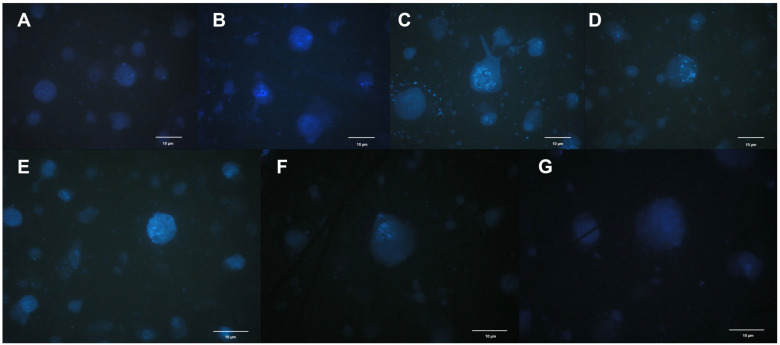
Nuclei degradation in the corona of cut ‘Dutch Master’ daffodils, collected from flowers (**A**) immediately after harvest, (**B**) 1 day after harvest, (**C**) 2 days after harvest, (**D**) 3 days after harvest, (**E**) 4 days after harvest, (**F**) 5 days after harvest, (**G**) 6 days after harvest.

The degree of degradation of the cell nuclei was also determined under a light microscope. A significant effect of flower developmental stage on the process of nuclei degradation was observed (Figure 2 and Figure 3). At the initial stage of flower development, i.e., on the day of harvest, when flowers were still in the bud stage, the nuclei were much less visible than they were in full bloom (Figure 2A and Figure 3A).

At full flower bloom (days 1–4), both in the perianth and the corona, the cell nuclei were clearly visible and well preserved in shape, and the nuclear envelope had not yet condensed (Figure 2B–D and Figure 3B–E). In addition, chromatin fragmentation was also observed in the corona (Figure 3D).

During the last stage—total loss of decorativeness, i.e., on days five and six—the cell nuclei were much less visible because of the progressive degradation process (Figure 2F,G and Figure 3F,G). On day four after flower harvest, the nuclei in the perianth began to change their spherical shape and decay (Figure 2E). In the corona, the nuclear envelope retained its continuity much longer and started to disintegrate later than in the perianth.

## 3. Discussion

According to Shibuya and co-workers [6], understanding the biochemical mechanisms during plant senescence, together with the morphological changes in petal PCD, is one of the major challenges. A consequence of the senescence process of cut flowers is the loss of decorativeness. Senescence is a process of degradation and remobilization of proteins, lipids, and nucleic acids, leading to cell death in an organ [7]. Earlier research by Rabiza-Świder et al. [1] showed that perianths generally have the most metabolic activity during flower opening, but this changes during senescence. In daffodils, the ovary becomes the most metabolically active floral structure where the sugar from the preservative is directed to [1]. Soluble carbohydrates are an essential energy source for cut flowers: they enhance petal growth and coloration, making them crucial for flower quality [19]. Typically, the vase life of flowers with relatively high sugar contents is relatively long, whereas that of flowers with relatively low sugar content is relatively short [20]. However, Eason et al. [21] reported that the petal sugar content does not always correlate with flower longevity. In cut daffodil flowers, a greater carbohydrate pool was detected in the perianth, which has a shorter vase life, than in the corona, which has a longer longevity. Rabiza-Świder et al. [1] suggested different roles of individual floral structures of daffodils in sugar translocation within the flower, which can be associated with turgor loss and the removal of water from the perianth faster than in the corona. The current study highlights a crucial connection between total sugars and free proline content: a higher sugar content reduced the free proline content, especially in coronas, which have a longer vase life than perianths.

In the daffodil flowers, the free proline content steadily increased from the bud stage to the complete loss of decorativeness. Kumar et al. [22] reported a significant increase in the endogenous proline content of cut rose flowers at the open stage, followed by a dramatic decrease as the petals senesced. The increase in proline content during senescence may be induced by a reduction in water potential, as demonstrated in rose petals [23]. This response is closely linked to the senescence process of cut flowers [24,25]. Increases in the free proline content depend on the species and even the cultivar. For instance, in *Clematis*, proline content increased by no more than 1.5-fold relative to the initial levels [24], whereas in cut roses, a substantially higher increase of up to 14-fold was observed [22]. The growth of peony is between 2-fold in ‘Gayborder June’ and 10-fold in ‘Festiva Maxima’ [26]. In the tested daffodil cultivars, the difference was also observed; in ‘Dutch Master’, there was 5-fold growth, and in ‘Mando’, there was, on average, 2.5-fold growth (2-fold in the perianth and 3-fold in the corona). Researchers have not fully explored whether increased proline content contributes to flower senescence or is a consequence of senescence [26]. According to Zhang and Becker [25], proline can be unleashed as a fuel source for ATP production during flower senescence. Parveen et al. [27] reported that proline acts as an efficient sentinel to impede various detrimental effects associated with petal senescence. In the present study, the free proline content was associated with the levels of various compounds, e.g., in perianths, high levels of proline corresponded with high levels of hydrogen peroxide, which is the primary reactive oxygen species, and vice versa in coronas: high levels of proline corresponded with lower levels of hydrogen peroxide. On the other hand, a strong positive correlation was observed between free proline content and catalase activity, which is the main enzyme that degrades hydrogen peroxide. Thus, it can be seen that an increase in proline, on the one hand, can cause the formation of free radicals, but at the same time, activate defense enzymes.

During senescence, lipid membrane peroxidation is induced, accompanied by alterations in membrane permeability, resulting in cellular sap leakage and ultimately leading to cell death [28]. The intermediate products of lipid peroxidation, including reactive free radicals, along with the end product malondialdehyde (MDA), accumulate progressively throughout senescence [29,30]. Zhao et al. [30] have identified these parameters as critical indicators reflecting the extent of cellular damage associated with plant senescence. In the present study, MDA content also correlated with the proline content, considered by many authors as one of the primary indicators of the senescence process. In cut ‘Dutch Master’ and ‘Mando’ daffodil flowers, the MDA content increased significantly, both in the perianth and corona, but in the perianth, the value was many times higher than in the corona. In the perianth of ‘Dutch Master’, the first significant growth occurred as early as 24 h after harvest, and in coronas, it occurred 24 h later. In ‘Mando’ flowers, the first increase in the MDA content occurred on the second day after harvest, both in the perianth and corona, but the value of the MDA content in the perianth was greater than that in the corona, similar to that on the 7th day after harvest (at the end of the senescence process). An increase in the MDA content was also observed in peony flowers [30]. In cut snapdragons, an increase in the MDA content was recorded, especially in older parts of inflorescences [12].

The hydrogen peroxide content in the perianth of ‘Dutch Master’ increased significantly, especially in the last two days, when visible damage was detected, reaching its highest value, which was 36% greater than that on the harvest day. The opposite was true for the corona, where the H_2_O_2_ content gradually increased and then decreased, reaching 44% lower content on the last day than at the beginning, which was 2.6 times lower than that in the perianth. In the ‘Mando’ perianth, the hydrogen peroxide content was the highest on the harvest day and on the last day, when the flowers experienced visible damage. In the corona, the highest level of H_2_O_2_ was detected on the harvest day and on the second and fifth days after harvest. On the sixth day, when the perianth had already lost its decorative value and the corona was still decorative, the level of hydrogen peroxide in the perianth leaves was noticeably greater. This study highlights significantly higher levels of hydrogen peroxide in the perianth than in the corona of daffodil flowers. The content of hydrogen peroxide is the primary reactive oxygen species that accumulates during the senescence of cut flowers [12,13,30,31]. Also, in the study of cut snapdragon flowers, the level of H_2_O_2_ was much higher in the physiologically older florets from the lower part of the inflorescences than that in the younger florets from the upper parts of the inflorescences [12]. In cut daylily flowers, an increase in the H_2_O_2_ content occurred before the flowers fully developed [13]. Hossain et al. [32] reported a gradual increase in H_2_O_2_ content during the senescence of sword lily, with the highest H_2_O_2_ content recorded when the flowers opened completely. Also, studies on cut orchids [31] and peony [30] flowers have shown that their senescence is related to an increase in H_2_O_2_.

An analysis of enzyme activity in the defense system against the reactive oxygen species catalase was also performed, revealing an increase in activity in both cultivars. The growth in plants subjected to oxidative stress was affected by part of the flower. Due to the toxic nature of reactive oxygen species (ROS), their efficient scavenging is essential for cellular viability. Given the inevitable production of oxygen free radicals and hydrogen peroxide during metabolic processes, plants have evolved an array of antioxidant systems to regulate and maintain intracellular ROS homeostasis [33]. An effective strategy to eliminate ROS is through the enzymatic activity of superoxide dismutase, catalase and ascorbate peroxidase [34]. Catalase transforms hydrogen peroxide into oxygen and water [19]. The highest catalase activity in the ‘Dutch Master’ perianth was recorded on the 6th day after harvest. In the corona, the highest level was recorded earlier, on the 3rd day after harvest. Similarly, in the ‘Mando’ perianth, the catalase activity increased by almost three times from the harvest day to the seventh day, whereas in the corona, the level on the seventh day was only 1,4 times greater than that at the beginning, but the greatest growth was observed earlier than that in the perianth on the second day after harvest. Earlier activation of enzymes in the coronas may have allowed earlier deactivation of hydrogen peroxide in the coronas than in the perianths. At the same time, the correlation analysis showed that in coronas, higher activity of antioxidant enzymes corresponds with lower content of hydrogen peroxide. In contrast, in perianths, high catalase activity does not correspond with low hydrogen peroxide levels. This shows that the antioxidant system works faster and more efficiently in the coronas than in perianths. In cut chrysanthemum and peony flowers, the activity levels of protective enzymes, including catalase, first increase but then decrease during the late vase period [30,35]. Similarly, in cut rose flowers, after the initial increase in catalase activity in younger flowers, a reduction in catalase activity was observed in older flowers [36]. According to Zhao et al. [30], catalase is an enzyme that is first activated to protect flowers from ROS damage when stress conditions induce ROS production [30].

One of the most important changes in petal senescence occurring during PCD is the formation of autophagosomal vacuoles, which increase their volume and ability to absorb other organelles, such as mitochondria, endoplasmic reticulum or ribosomes; fragmentation of the cell nucleus and cytoplasm by the action of endonucleases and proteinases; and destruction of cell membranes due to the breakdown of lipids [8,9].

During PCD in plant cells, the nuclei undergo various morphological changes, including chromatin condensation and nuclear fragmentation [6]. The degree of degradation of cell nuclei was noted in cut daffodil ‘Dutch Master’ flowers. The flower developmental stage significantly affects this process. Wagstaff et al. [9] reported in Peruvian lily flowers that degradation of the nucleus and petal cells begins in the fully opened flower and that PCD processes are triggered very early and continue throughout the opening of the flower until the end of the flowering period. Research on PCD in the petals of model plants has shown that this process begins at a very early stage of development, still in the bud phase [8,9,13]. In daffodils at the bud stage, i.e., on the day of harvest, the nuclei were much less visible than they were in full bloom. At full flower bloom, both in the perianth and the corona, the cell nuclei were clearly visible and well preserved in shape, and the nuclear envelope had not yet condensed. In addition, chromatin fragmentation could also be observed in the corona. In the last stage—total loss of decorativeness—the cell nuclei were much less visible due to the progressive degradation process. On the 4th day after flower harvest, the nuclei in the perianth began to change their spherical shape and decay. In the corona, the nuclear envelope retained its continuity much longer and started to disintegrate later than in the perianth. This is possibly because the corona has a longer vase life than the perianth [1].

Chromatin condensation is initiated at the nuclear periphery and subsequently propagates throughout the entire nucleus, reflecting a spatially coordinated reorganization of the chromatin architecture [6]. In *Ipomoea* petals, chromatin condensation in the entire nucleus also results in a reduction in the size of the nucleus [37]. Chromatin condensation has been described in senescing petals of snapdragon, *Argyranthemum*, *Petunia* [14], *Freesia* [38], *Gladiolus* [39], and *Ipomoea* [37].

Nuclear fragmentation has rarely been reported in plant cells, especially petals. According to Shibuya et al. [6], “at least two contrasting nuclear morphologies have been observed during plant PCD. One is nuclear fragmentation, and the other is fragmentation of chromatin inside the nucleus.” However, they claim that, “it is not yet clear whether morphological changes in the nucleus during plant PCD can be classified into two categories” [6].

In conclusion, flower death is the end result of a process called flower senescence, and this period is genetically programmed [3].

## 4. Materials and Methods

Flowers of Division 1—Trumpet daffodil (*Narcissus* L.) cultivars [40], ‘Dutch Master’ and ‘Mando’—were harvested in the morning, in the gooseneck bud stage, when the buds were tilted from vertical to horizontal (flowers at 90–120° angles from the stem) [17]. They were immediately transferred to the laboratory, trimmed to 30 cm as measured between the scape base and its bend, and placed into vases with water, which was not exchanged during the experiment, but it was replenished as needed.

The experiments were carried out in a room with a controlled temperature of 20 °C ± 1 °C, a relative humidity of 60%, and a quantum irradiance of 35 μmol·m^−2^·s^−1^ (fluorescent lamp LF80 36 W/850, Natural Daylight, Pila) under a 12 h day/12 h night regime.

The senescence parameters were determined separately for the perianth and corona. Samples were collected each day, starting from the day of harvest until day 6 for ‘Dutch Master’ and day 7 for ‘Mando’ after harvest. For each analysis, on each measuring date, six flowers were taken. The flower tissue from perianths and coronas, separately, was finely cut and evenly mixed, and three samples of 0.25 g each were taken. Three determinations were made for each sample, resulting in a total of nine readings for each data average. Additionally, three samples were taken for dry weight determination: the plant material was dried at 105 °C until a constant weight was achieved. The results were subsequently calculated on a dry matter basis (DW).

### 4.1. Biochemical Assays

Total sugars were measured as described by Dubois et al. [41] and expressed in mg glucose·g^−1^ dry weight (DW). The material was homogenized in 80% ethanol. The extracts were incubated for 20 min in a boiling water bath with 5% phenol and 96% H_2_SO_4_, and the absorbance was measured at 490 nm. The total sugar content was calculated from a previously plotted standard curve prepared for glucose.

The reducing sugar content was measured via the Somogyi method as modified by Nelson [42] and expressed in mg glucose·g^−1^ dry weight (DW). The material was homogenized in 80% ethanol. The extracts were incubated for 20 min in a boiling water bath with copper reagent; molybdenum arsenic reagent was added, and the absorbance was measured at 520 nm. The reducing sugar content was calculated from a previously plotted standard curve prepared for glucose.

The free proline content was determined according to Bates et al. [43] by measuring the quantity of a colored reaction product of proline with ninhydric acid. The absorbance was read at 520 nm. The amount of proline was calculated from a previously plotted standard curve and expressed in µmol·g^−1^DW.

Malondialdehyde (MDA) was measured according to Hodges et al.’s method [44] on the basis of the color reaction with thiobarbituric acid, and its values are given in nmol·g^−1^DW.

The hydrogen peroxide (H_2_O_2_) content of the petals was measured spectrophotometrically after the reaction with potassium iodide (KI) as described by Jędrzejuk et al. [45]. The H_2_O_2_ content was expressed at 390 nm as µg of hydrogen peroxide per g on a dry weight basis.

The catalase (CAT) activity (EC 1.11.1.6) was determined spectrophotometrically as the rate of H_2_O_2_ disappearance at 405 nm according to Goth [46] and expressed as mcatals per g DW.

### 4.2. Nuclear Morphology

For observations of nuclear morphology and chromatin condensation, nuclei from the perianth or coronas of cut ‘Dutch Master’ daffodil flowers were isolated according to the methods of Galbraith et al. [47]. Isolated nuclei were stained with 4′,6-diamidine-2′-phenylindole dihydrochloride (DAPI; Sigma-Aldrich, St. Louis, MO, USA) by a 5 min pretreatment with 0.2 mol dm^−3^ citric acid and a 30 min incubation with DAPI (2 mg·dm^−3^) in PBS, pH 7.4. DAPI staining is used to visualize cell nuclei by binding strongly to DNA. The protocol involves fixing and permeabilizing cells to allow DAPI to enter and bind DNA, enabling fluorescent detection under UV light. The microphotographs used to visualize the morphological changes in the nuclei and nuclear chromatin were obtained with an AX Provis fluorescence microscope (Olympus, Tokyo, Japan) with Quick Photo Pro software Version 3.0.

### 4.3. Statistical Analyses

The results were statistically evaluated via ANOVA 1 via the IBM^®^ SPSS^®^ Statistics 29 program. Duncan’s test at α = 0.05 was applied to assess the significant differences between the means.

The Pearson correlation coefficients between the content of total sugars, reducing sugars, free proline, malondialdehyde, hydrogen peroxide and catalase activity in the perianth and corona of ‘Dutch Master’ and ‘Mando’ daffodils were examined separately. The Pearson correlation was conducted with the use of SPSS (IBM, Armonk, NY, USA). Correlation significance at *p* < 0.05 was determined (0.1–0.29—very low; 0.30–0.49—low; 0.50–0.69—restrained; 0.70–0.89—high; >0.90—very high) [48].

## 5. Conclusions

This manuscript presents a comparison of the senescence process in perianths and coronas of cut daffodil flowers, based on various biochemical and structural parameters (carbohydrates, free proline, malondialdehyde contents, hydrogen peroxide levels, catalase activity, and nuclei degradation), which have not been previously reported. During the senescence of cut daffodil flowers, increases in free proline, malondialdehyde and hydrogen peroxide contents and increased catalase activity were observed. They occurred faster in the perianth than in the corona, excluding carbohydrates. One of the symptoms of daffodil flower senescence was the degradation of cell nuclei. The nuclei in the perianth began to change their spherical shape and decay earlier than in the corona. Faster senescence-related changes in the perianth translate into a shorter vase life for this part of the flower.

## Figures and Tables

**Table 1:** Changes in the values of selected parameters in daffodil ‘Dutch Master’ flowers placed after cutting into water.

Content/Activity	Part of the Flower	Term (Date)						
		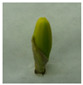	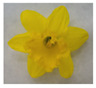	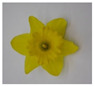	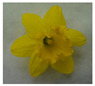	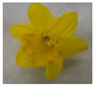	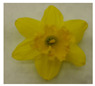	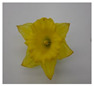
		Harvest Day	1	2	3	4	5	6
total sugars [mg·g^−1^DW]	perianth	B 485.4 c	A 413.0 b	A 422.9 b	A 404.2 b	A 328.9 a	B 460.0 c	B 566.3 d
	corona	A 436.2 a	B 484.6 b	B 567.9 c	B 647.3 d	B 546.0 c	A 412.5 a	A 382.3 a
reducing sugars [mg·g^−1^DW]	perianth	A 150.2 a	A 269.5 b	A 317.1 e	A 296.6 de	A 256.5 b	A 306.8 e	A 279.0 bc
	corona	A 132.8 a	B 300.0 c	A 307.1 c	A 293.9 c	A 230.0 b	A 284.4 c	A 268.3 bc
free proline [µmol·g^−1^DW]	perianth	A 1.93 a	A 2.74 b	A 3.97 c	A 4.83 d	A 4.24 cd	B 7.69 e	A 11.36 f
	corona	A 1.89 a	A 2.85 b	A 4.11 c	B 5.49 d	B 5.13 e	A 6.80 f	A 11.12 g
malondialdehyde MDA [nmol·g^−1^DW]	perianth	B 121.0 a	B 127.9 b	B 139.4 cd	A 133.0 bc	A 108.3 a	B 147.8 d	A 137.9 c
	corona	A 109.8 a	A 101.0 a	A 126.6 c	B 147.0 e	A 114.2 b	A 139.8 de	A 136.4 d
hydrogen peroxide [μg·g^−1^DW]	perianth	A 1.63 ab	B 1.73 bc	B 1.42 a	B 1.54 ab	B 1.64 ab	B 1.91 c	B 2.22 d
	corona	A 1.58 d	A 0.85 a	A 1.14 bc	A 0.97 ab	A 0.74 a	A 1.32 c	A 0.85 a
catalase [mcatal·g^−1^DW]	perianth	B 1143.1 a	A 1398.1 a	B 2764.2 b	A 1373.3 a	A 1312.3 a	A 3194.2 b	B 5319.1 c
	corona	A 569.7 a	B 2817.6 c	A 1544.1 b	B 4338.7 e	B 4190.8 e	A 3480.5 d	A 4678.7 e

Means in the rows followed by the same lowercase letter do not differ significantly at the probability level α = 0.05 (Duncan’s test). Lowercase letters describe the effect of date on selected parameters in the perianth or corona. Means in columns followed by the same capital letter do not differ significantly at the probability level α = 0.05 (Duncan’s test). Capital letters describe the effect of part of the flower on selected parameters on the respective measurement dates. Statistical analyses were performed separately for the parameter under study. DW—dry weight.

**Table 2:** Changes in the values of selected parameters in daffodil ‘Mando’ flowers placed after cutting into water.

Content/Activity	Part of the Flower	Term (Date)							
		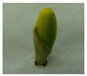	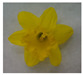	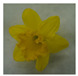	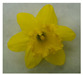	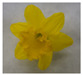	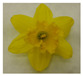	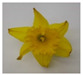	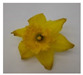
		Harvest Day	1	2	3	4	5	6	7
total sugars [mg·g^−1^DW]	perianth	B 437.0 bc	B 466.8 d	A 322.6 a	B 415.4 bc	B 444.3 cd	A 399.9 b	B 339.1 a	B 345.3 a
	corona	A 338.8 b	A 400.4 c	B 347.8 bc	A 281.3 b	A 342.1 bc	A 381.9 c	A 261.4 a	A 181.1 a
reducing sugars [mg·g^−1^DW]	perianth	A 182.9 b	A 142.3 a	A 256.2 d	B 372.2 f	B 339.2 e	B 348.7 ef	B 234.6 cd	B 215.5 c
	corona	A 186.9 de	A 120.6 a	A 239.2 f	A 197.5 e	A 178.9 de	A 171.9 cd	A 152.5 bc	A 137.7 ab
free proline [µmol·g^−1^DW]	perianth	B 3.15 b	A 2.51 a	A 2.60 a	B 5.52 c	B 8.64 e	B 7.47 d	B 9.29 f	A 9.81 f
	corona	A 2.46 a	A 1.99 a	B 3.19 b	A 4.61 c	A 6.35 d	A 6.21 d	A 8.10 e	B 11.13 f
malondialdehyde MDA [nmol·g^−1^DW]	perianth	B 142.0 b	B 147.9 b	B 159.4 cd	A 153.0 bc	A 128.3 a	B 167.8 d	A 159.9 cd	B 175.2 d
	corona	A 129.5 a	A 121.0 a	A 146.3 c	B 168.1 e	A 134.1 b	A 159.9 de	A 154.4 d	A 160.2 d
hydrogen peroxide [μg·g^−1^DW]	perianth	B 2.66 b	B 2.03 a	A 2.05 a	B 2.12 a	A 1.95 a	B 2.36 ab	B 1.97 a	B 2.68 b
	corona	A 2.24 c	A 1.61 ab	A 2.19 c	A 1.57 ab	A 1.72 b	A 2.04 c	A 1.35 a	A 1.72 b
catalase [mcatal·g^−1^DW]	perianth	A 1073.1 bc	A 751.7 a	A 894.4 ab	A 1109.4 c	A 979.5 bc	A 1081.8 bc	A 1337.9 d	A 2786.7 e
	corona	B 1693.0 b	A 716.7 a	B 2974.7 d	B 1799.6 b	B 2069.6 bc	B 1797.8 b	B 2128.4 bc	A 2383.9 c

Means in the rows followed by the same lowercase letter do not differ significantly at the probability level α = 0.05 (Duncan’s test). Lowercase letters describe the effect of date on selected parameters in the perianth or corona. Means in columns followed by the same capital letter do not differ significantly at the probability level α = 0.05 (Duncan’s test). Capital letters describe the effect of part of the flower on selected parameters on the respective measurement dates. Statistical analyses were performed separately for the parameter under study. DW—dry weight.

## Data Availability

Data are contained within the article.

## Abbreviations

The following abbreviations are used in this manuscript:

DW	Dry Weight
MDA	Malondialdehyde
PCD	Programmed Cell Death
